# Ginsenoside Rg1 in Parkinson’s disease: from basic research to clinical applications

**DOI:** 10.3389/fphar.2025.1490480

**Published:** 2025-04-16

**Authors:** Qianyan Wang, Lei Wei, Guanghui Chen, Qiang Chen

**Affiliations:** ^1^ Liyuan Cardiovascular Center, Liyuan Hospital, Tongji Medical College, Huazhong University of Science and Technology, Wuhan, China; ^2^ Department of Pharmacy, Tianmen Hospital of Traditional Chinese Medicine Affiliated to Hubei University of Chinese Medicine, Tianmen, China; ^3^ Department of Pharmacy, Renmin Hospital, Wuhan University, Wuhan, China; ^4^ Department of Pharmacy, Liyuan Hospital, Tongji Medical College, Huazhong University of Science and Technology, Wuhan, China

**Keywords:** ginsenoside Rg1, Parkinson’s disease, anti-inflammatory, antioxidative, neuroprotection

## Abstract

This review provides an in-depth exploration of the potential of Ginsenoside Rg1 in the treatment of Parkinson’s disease (PD). The emphasis of this article was the therapeutic mechanisms of Rg1, which involved the reduction of inflammation, antioxidant properties, support for neuronal survival and regeneration, regulation of cellular energy processes, and enhancement of autophagic pathways. Rg1 may protect neurons and improve both motor and cognitive impairments associated with PD through multiple mechanisms. However, challenges exist in the clinical application of Rg1, such as low bioavailability as well as a lack of comprehensive long-term safety and efficacy data. This article also reviewed network pharmacology analyses published previously to identify and explore the potential molecular targets of Rg1 in PD treatment, while evaluating strategies such as drug delivery technologies, optimizing administration routes, and combination therapies. Ultimately, this review highlights the necessity for large-scale clinical trials to validate the clinical efficacy of Rg1 and discusses its potential for PD treatment clinically.

## Introduction

Parkinson’s disease (PD) is a long-lasting neurodegenerative condition that manifests mainly as motor dysfunction (Ragothaman et al., 2023). The disease is characterized by the degeneration of dopaminergic neurons in the substantia nigra and the formation of Lewy bodies, which are linked to the abnormal accumulation of α-synuclein protein (Becerra-Calixto et al., 2023). Patients with PD may experience resting tremor, bradykinesia, muscle rigidity, and postural instability in most cases (Asci et al., 2023). The occurrence and prevalence of PD are on the rise in the context of the global increase in the elderly population (Ou et al., 2021). PD may affect about 1% of individuals aged 60 and older, with the prevalence increasing to 3%–4% in those aged 80 and above, showing a higher prevalence in men than women as well (Song et al., 2022). Current therapeutic option usually includes dopamine replacement therapy to provide the effect of symptom relief. However, this therapy is impossible to prevent disease progression and may result in side effects such as dyskinesia and the “wearing-off” phenomenon after long-term use (Mishima et al., 2023), causing serious impact on the quality of life of patients (Simonet et al., 2022). The potential of natural products has been highly concerned in treating neurodegenerative diseases recently. Panax ginseng, a well-known herbal remedy, has been confirmed to have active constituents called ginsenosides, supporting its various pharmacological benefits. Based on their chemical structure, ginsenosides are divided into two main categories: protopanaxadiol-type (e.g., Rb1, Rb2, Rc, and Rd) and protopanaxatriol-type (e.g., Re, Rg1, Rg2, and Rh1) (Zhang et al., 2024; Roos et al., 2022; Jin et al., 2019). These compounds display a broad range of biological activities (Table 1). Among these, ginsenoside Rg1 has gained particular attention due to its distinct neuroprotective effects. Rg1 stands out from other ginsenosides because it exhibits an extensive mechanism of preventing inflammation and oxidation as well as its modulation of key signaling pathways. Based on the specific properties of Rg1, it may be feasible for alleviating or retarding PD symptoms. The efficacy of Rg1 in experimental settings together with its safe application profile generates scientific interest in this compound. The variety and strength of the mechanisms of Rg1 establish it as the perfect candidate for additional research as a PD therapy.

**TABLE 1:** Ginsenosides and their effects.

Ginsenoside type	Molecular formula	Effects	References
Rg1	C42H72O14	Alleviate traumatic brain injury, prevent cognitive impairment, cardioprotective effects, Alzheimer’s disease (AD), Parkinson’s disease (PD), treatment of inflammatory bowel disease and diabetes, reduce depression, prevent cellular aging, alleviate liver injury, improve reproductive function damage, prevent post-traumatic stress disorder (PTSD), Huntington’s disease (HD), reduce glomerular fibrosis, and promote sleep	Liu et al. (2023)
Rb1	C54H92O23	Anti-angiogenesis, anti-inflammatory, cardioprotective, anti-aging, anti-diabetic, antidepressant and neuroprotective effects, prevention of alcoholic liver disease	Wang et al. (2022)
Re	C48H82O18	Anti-diabetic effects, inhibit neoplasm formation, alleviate cognitive deficits, and reduce myocardial injury	Sarhene et al. (2021)
Rd	C48H82O18	Reduce lung injury, alleviate immune neuroinflammation, prevent cardiovascular diseases, improve auditory cortex damage, reduce anxiety/depression, and alleviate obesity	Wang L. et al. (2023)
Rb2	C53H90O22	Hematopoietic, neuroprotective and anti-inflammatory effects, improve platelet function, reduce obesity, and treat colon cancer	Sun et al. (2022)
Rc	C53H90O22	Cardioprotective and anti-inflammatory effects	Ren Z. et al. (2023)
Rb3	C53H90O22	Cardioprotective effects, reduce lung and kidney injury	Madhi et al. (2021)
Rf	C42H72O14	Anti-melanogenesis, anti-depressant, anti-inflammatory and anti-nociceptive effects	Lu et al. (2022)
Rg2	C42H72O13	Alleviate brain injury, affect cell growth, improve memory impairment and neuronal death, exert anti-arrhythmic effects, and reduce lipid formation	Shi et al. (2022)
Rg3	C42H72O13	Alleviate heart failure, exert anti-inflammatory effects, reduce skin inflammatory diseases, alleviate osteoporosis, exert anti-angiogenesis effects, reduce endothelial dysfunction, prevent atherosclerosis, and inhibit liver cancer and gastric cancer	Liang et al. (2022)
Rh1	C36H62O9	Immunomodulation, improve diabetic nephropathy and alleviate cognitive deficits	Ding et al. (2023)

Rg1 represents a triterpenoid saponin compound which falls under the protopanaxatriol group of ginsenosides. The molecule Rg1 consists of C42H72O14 atoms and has a molecular weight of 801.01 (Choi et al., 2022). Rg1 features a sugar chain bonded to the 3-hydroxy location on its triterpenoid core structure, which usually includes glucose and rhamnose units (Yang et al., 2022; Zhang H. et al., 2021). Rg1 demonstrates outstanding water solubility and effective compatibility with polar organic solvents such as ethanol, owing to the existence of multiple hydroxyl groups in its structure. The compound maintains chemical stability, yet with thermal and acidic resistance, while being susceptible to alkaline hydrolysis (He et al., 2021; Wang et al., 2024). Rg1 stands out in scientific research because of its powerful capabilities to fight neuroinflammation, produce antioxidant effects, and support neuronal survival, while promoting regeneration and energy metabolism regulation as well as its impact on autophagic processes (Li et al., 2023; Ji et al., 2023; Yao et al., 2021). Rg1 achieves neuroprotection by blocking pro-inflammatory cytokine release while activating signaling pathways that support synaptic plasticity and simultaneously promoting both neuronal survival and regeneration. Rg1 has been reported to effectively reduce PD pathology across different biological levels, with confirmed therapeutic potential in animal experiments. The underlying mechanisms of Rg1 have proven essential to reduce symptoms and delay disease development while enhancing life quality for PD patients. Accordingly, the present article reviews current findings about the effectiveness of Rg1 in PD treatment, as well as its anti-inflammatory and antioxidative properties along with neuroprotective mechanisms. This study aims to establish a research framework and suggest clinical usage improvements.

## Inhibition of neuroinflammation

Neuroinflammation may be triggered by the excessive activation of microglia and astrocytes, serving as a fundamental characteristic of PD. The excessive activation of immune cells in the brain produces inflammatory molecules TNF-α, IL-1β and IL-6, worsening damage to dopamine-producing neurons and speed up PD development (Chiu et al., 2023; Zhang et al., 2022). Rg1 can suppress inflammatory responses by blocking essential inflammatory pathways such as NF-κB, MAPK, JAK2/STAT3, and TLR4/NF-κB, leading to the production and release of decreased inflammatory mediators (Im, 2020). Rg1 demonstrates anti-inflammatory properties, which can protect nerve cells and significantly improve PD motor symptoms such as bradykinesia and rigidity, as well as inflammatory-related symptoms (Zhou et al., 2025).

The management of PD relies heavily on anti-inflammatory effects, highlighting the necessity for deciphering the mechanism of oxidative stress substantially driving disease progression. We will analyze the therapeutic mechanism of Rg1 in PD treatment through its strong antioxidant properties, which extend therapeutic advantages beyond basic protection.

## Antioxidation

Oxidative stress plays a central role in the development of PD, primarily through the excessive accumulation of free radicals and reactive oxygen species (ROS), thereby leading to neuronal damage and cell death (Mittal et al., 2023; Jiang and Bai, 2022). Rg1, recognized for its strong antioxidant properties, exerts its protective effects via several molecular mechanisms (Boas et al., 2021). Specifically, it effectively counteracts the harmful effects of free radicals by enhancing the activity of antioxidant enzymes, including superoxide dismutase (SOD) and glutathione peroxidase (GSH-Px), thus alleviating neurotoxicity induced by oxidative stress (Sun et al., 2022). Rg1 boosts cellular antioxidant defenses through activating Nrf2/ARE pathway, further promoting the expression of key antioxidant genes such as heme oxygenase-1 (HO-1) and NAD(P)H: quinone oxidoreductase 1 (NQO1) (Zhang et al., 2016). Moreover, Rg1 can also prolong its neuroprotective effects by preventing lipid peroxidation, particularly the formation of malondialdehyde (MDA) to protect cell membrane integrity (Zhang et al., 2023; Hong et al., 2022). Through the synergistic actions of these antioxidant and anti-inflammatory pathways, Rg1 plays a pivotal role in alleviating motor symptoms (e.g., bradykinesia and resting tremors), which may contribute to slowing the progression of PD.

The treatment of PD must emphasize on neuron survival and regeneration promotion after defending neurons against oxidative and inflammatory damage. Rg1 demonstrates strong potential to encourage neuronal healing by activating multiple signaling pathways, which will be subjected to in-depth investigations in the next section.

## Promotion of neuronal survival and regeneration

By activating the Brain-derived neurotrophic factor/tropomyosin receptor kinase B (BDNF-TrkB) signaling pathway, Rg1 can protect neurons against degeneration while controlling synaptic plasticity (Zhong et al., 2020). The increased BDNF levels may further trigger the activation of phosphoinositide 3-kinase/protein kinase B (PI3K/Akt) pathway, serving as a key mechanism in neuronal development and survival (Hannan et al., 2020). The pathway promotes cell survival while preventing apoptosis. Simultaneously, the Mitogen-activated protein kinase/extracellular signal-regulated kinase (MAPK/ERK) pathway controls cell growth and differentiation (Ma et al., 2016). Rg1 exhibits neuroprotective properties by blocking neuronal apoptosis through its suppression of Caspase-3 activity (Fan et al., 2018). The neuroprotective properties of Rg1 extend to neuronal repair considering its modulatory role in Glycogen synthase kinase 3 beta (GSK-3β) and activation of the Wnt signaling pathway, thereby improving motor function and decelerating PD progression (Yang et al., 2022).

The therapeutic results for PD treatment improve when neuronal survival and regeneration receive support through optimized energy metabolism and enhanced autophagic processes. Here, we will elucidate the mechanism of action of Rg1 in PD through cellular energy balance improvement and autophagy stimulation to achieve better treatment results.

## Regulation of energy metabolism and enhancement of autophagy

**FIGURE 1:**
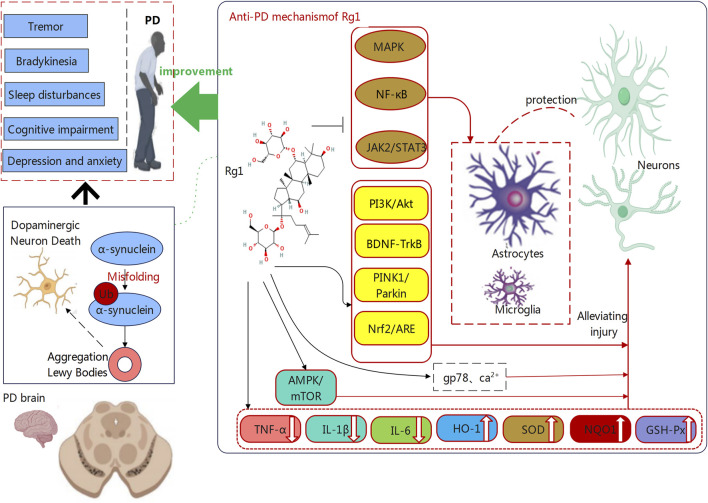
The mechanisms through which Ginsenoside Rg1 counteracts Parkinson’s Disease (PD). PD is characterized by α-synuclein aggregation and dopaminergic neuron degeneration. Rg1 protects neurons by reducing neuroinflammation and oxidative stress via the regulation of pathways such as NF-κB, MAPK, and Nrf2/ARE. It also enhances neuronal survival through PI3K/Akt, BDNF-TrkB, and PINK1/Parkin pathways. On these basis, Rg1 may alleviate PD symptoms such as tremor, bradykinesia, and cognitive impairment.

Beyond its previously identified benefits, Rg1 may be useful for treating PD through its regulation of energy metabolism and autophagic processes. In addition to protecting energy metabolism from high glucose disruption, this compound can also reduce mitochondrial damage and maintain mitochondrial membrane potential through ROS suppression and mitophagy stimulation (Yang et al., 2022; Ren Y. et al., 2023). On this basis, it can sustain neuronal survival and functional maintenance, while boosting cognitive performance, leading to improved quality of life for PD patients (Walter et al., 2016; Wang W. et al., 2023). Furthermore, Rg1 activates autophagy through the AMPK/mTOR signaling pathway to reduce neurodegenerative effects from metabolic disorders. The essential process of autophagy targets damaged cellular parts for degradation and removal, while maintaining neuronal health by eliminating damaged organelles and proteins (Ktena et al., 2022). Consequently, Rg1can support cellular homeostasis to prevent neuronal damage and death from progressing. The mechanism functions to protect motor abilities, which may benefit the alleviation of motor impairments in patients with PD (Shen et al., 2021). Rg1 demonstrates significant therapeutic promise for PD, explained by its multiple biological mechanisms, as shown in Figure 1. The use of Rg1 can integrate anti-inflammatory and antioxidative neuroprotection with metabolic regulation and autophagy enhancement, demonstrating therapeutic effectiveness. Meanwhile, Rg1 exhibits multiple effects cover a broad spectrum, thus revealing potential for PD treatment. These mechanisms improve motor functions while delivering significant benefits to cognitive performance. Our subsequent section examines the multiple pathways through which Rg1 improves learning and memory functions in PD patients.

## Improvement of learning and memory function in PD by Rg1

In the treatment of PD patients, Rg1 produces substantial improvements in cognitive functions (including learning and memory) through the activation of multiple biological pathways. Generally, PD shows major pathology through dopaminergic neuron degeneration in the substantia nigra, causing an approximately 80% decline of dopamine levels in the striatum (Liu J. Q. et al., 2020; Choi et al., 2021; Baudonnat et al., 2013). Dopamine loss may disrupt the motor control, while inducing severe cognitive function impairment, particularly disturbance of learning and memory functions (Silveira et al., 2019). Rg1 has been shown to support dopaminergic neuron survival and functional recovery, also increasing dopamine secretion and cognitive function improvement (Hernández-Vara et al., 2020). Its effect on increasing dopamine production and release can be explained by the activation of the PI3K/Akt signaling pathway to prevent neuronal apoptosis (Zhou et al., 2016). Consistently, this mechanism has been verified in multiple PD models, demonstrating Rg1’s capability to both maintain and elevate brain dopamine levels (Park et al., 2021).

Glutamate represents the dominant excitatory neurotransmitter across central nervous system pathways. As a result, excessive glutamate release may promote its role as a neurotoxin, causing damage to the function of neurons. PD patients commonly show cognitive deficiencies when glutamate levels rise above normal levels (Pourhamzeh et al., 2022). Rg1 protects neuronal functions from neurotoxicity by blocking excessive glutamate receptor activity and lessening glutamate release (Bacqué-Cazenave et al., 2020). Simultaneously, by targeting NMDA receptor activities, Rg1 improves memory-related tasks, while reducing glutamate discharge (Wang et al., 2016). Rg1 defends neurons against damage by reducing oxidative stress alongside glutamate-induced inflammation (Neshan et al., 2020).

Furthermore, Rg1 has been confirmed to raise BDNF levels, while triggering TrkB receptor activation to initiate PI3K/Akt and MAPK/ERK signal transmission. This agent affects the expression levels of essential synaptic plasticity-related genes and proteins. The behaviors at hand can strengthen synaptic interactions, while boosting connections in networks of neurons alongside both neurogenesis and synaptogenesis (Magistrelli et al., 2020; Thiruvengadam et al., 2021). Neural plasticity, or neural plasticity, is a process that involves structural and functional brain changes which occur due to learning experiences to support essential memory formation and storage processes. Prior experimental research has revealed the properties of Rg1 in amplifying neural plasticity capabilities (Feng et al., 2022).

A previous animal research has documented significant effect of Rg1 in improving cognitive function for PD models (Zhang W. et al., 2021). In another 6-OHDA-induced rat model with PD, Rg1 successfully elevated dopamine levels, leading to improved spatial learning and memory with enhanced synaptic plasticity effects (Massari et al., 2021). Animal study related to PD has proven the role of Rg1 as a potential therapeutic for neuroprotective and cognitive function improvements (Jiang et al., 2023). In AF64A memory deficit experiment, Rg1 profoundly improved cognitive functionality, exhibiting therapeutic potential for improving cognitive symptoms of PD. Y. R. Yan et al. also demonstrated in their study that PD patients experienced appreciable cognitive enhancements when treated with Rg1, accompanied by marked benefits in memory function and attention capabilities (Jagaran and Singh, 2022; Yan et al., 2021). Scientists connect the therapeutic improvement to Rg1 since this compound can increase dopamine and BDNF levels, whiledecreasing neuroinflammation, as well as supporting neuronal healing and growth (Huang et al., 2023). In addition, J. Prasuhn et al. reported that Rg1 increased spine levels of main neurotransmitters and neurotrophic factors, leading to both cognitive aid to PD patients and extensive brain protection (Prasuhn et al., 2021; Cifuentes et al., 2023).

Besides dopamine and glutamate targets, Rg1 interacts with multiple neurotransmitter pathways, including both acetylcholine and serotonin, which improves brain abilities, particularly learning and memory. Rg1 can promote the release of acetylcholine, a neurotransmitter maintaining critical cognitive functions, to enhance the cholinergic system, further bolstering cognitive capability (Liu Q. Q. et al., 2020; Singh et al., 2024; Sun et al., 2023). Through its effect on serotonin receptors, Rg1 brings two beneficial results, featuring both advanced cognitive performance and improved emotional health alongside mood (Gupta et al., 2021; Kiran et al., 2022; Khatri et al., 2023). Rg1 combats learning and memory deficits in PD by balancing dopaminergic and glutamatergic pathways, as well as raising BDNF amounts, and improving synaptic plasticity. Both animal and human investigations have shown that Rg1 significantly increases brain function, making it a feasible option for PD treatment.

So far, clinical trials on Rg1 are required to confirm its positive effects discovered during preclinical testing. The following section will analyze clinical research status on Rg1, while addressing difficulties and recommending strategies for moving beyond experimental research toward clinical practice.

## Clinical applications of Rg1 in PD

Current clinical research remains inadequate to determine the value of Rg1 in the treatment of PD. Research to date shows Rg1 safety and general tolerance. However, extended clinical trials are still needed to examine its therapeutic potential and functional mechanisms. Recently, the medical utility of Rg1 is expected to be upgraded through nanotechnology integration, so as to increase its bioavailability while targeting destinations more precisely. Nanomedicine techniques are beneficial for extending medicinal stability by enhancing the physicochemical characteristics of drugs, while improving their therapeutic effectiveness in real-world conditions. The design of Rg1 nanoparticles enables enhanced blood-brain barrier permeability, facilitating targeted treatment to affected regions, thus improving the therapeutic outcomes (McLoughlin et al., 2024). Current studies focus mainly on the immediate effect, while presenting scarce information about Rg1 as well as its long-term safety and effectiveness. In the future, extended observational studies are necessary to assess long-term side effects and benefits together with risk factors. Future long-term clinical trials should investigate effects of Rg1 on PD patients’ motor capabilities and cognitive functions as well as their non-motor symptoms (Lyu et al., 2022; Khattab et al., 2021). Subsequent scientific exploration needs to target advanced delivery systems to maximize Rg1 bioavailability. Moreover, additional attention can be paid to delivery methods using polymer microsphere and gel systems alongside transdermal patches together with other bypass for first-pass metabolism systems, thus enhancing both the absorption and efficacy for Rg1 treatment. An ongoing study proposes a notable method of using intranasal delivery to target brain tissues, where it appears to both increase CNS drug levels and improve medical outcomes (Parkash et al., 2024; Pires et al., 2022). Meanwhile, it is important to decipher the mechanism of Rg1 when used with other treatments to increase its effectiveness as a feasible therapy for PD. Rg1, combined with dopamine replacement therapies delivers, was found to enhance neuroprotection, while protecting remaining dopaminergic neurons through the ability to reduce L-DOPA doses, thereby minimizing treatment-related side effects (García-Cárceles et al., 2022). Rg1 presents promising clinical outcomes for PD, although its long-duration safety and effects should be established through further research. Collectively, future wide-ranging clinical trials and multiple center research endeavors are essential to confirm the therapeutic benefit of Rg1 and its potential combined treatments for PD, which can facilitate the formulation of better treatment options for patients with PD.

## Network pharmacology analysis

**FIGURE 2:**
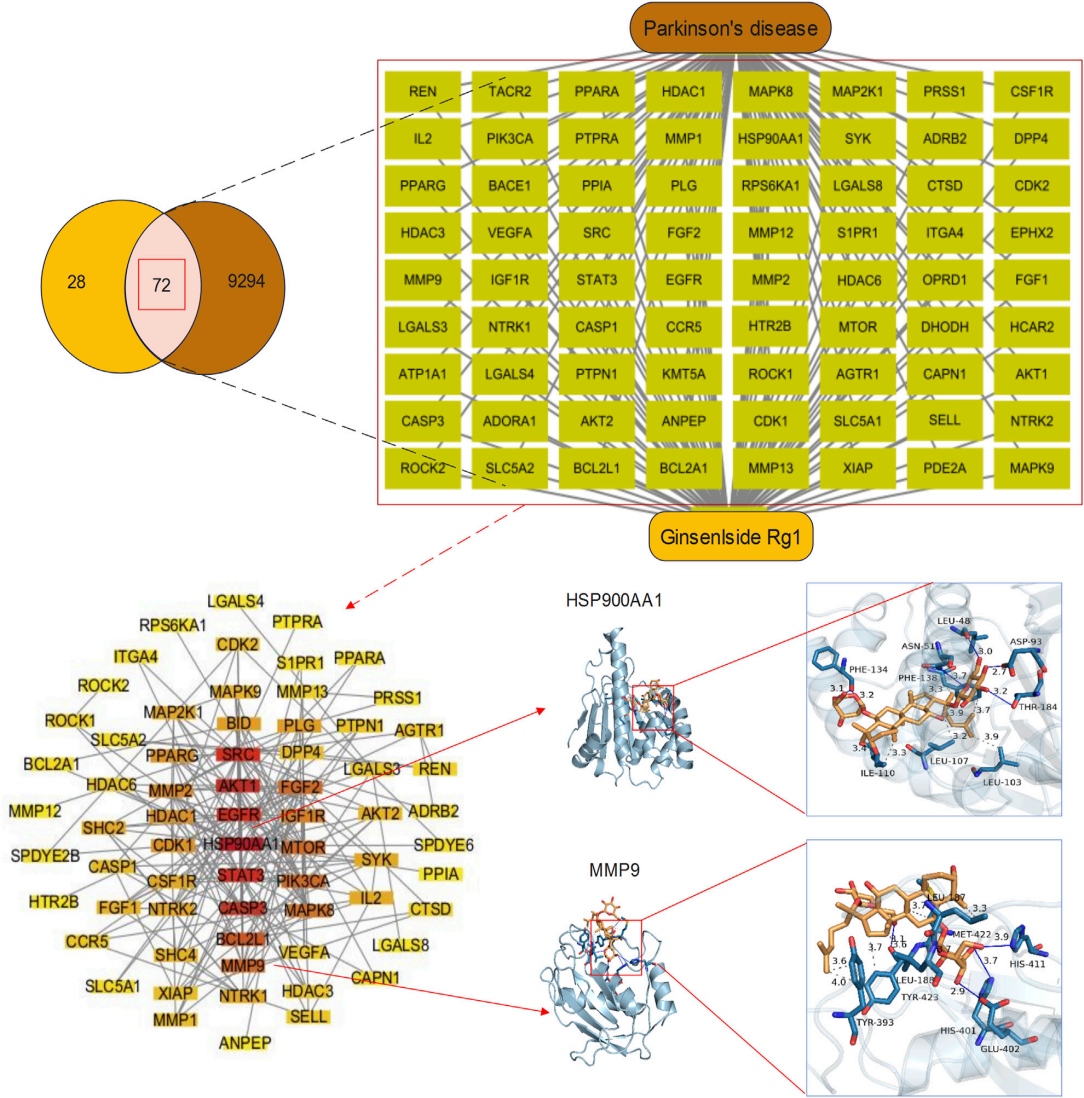
Potential targets and molecular docking of Rg1 against PD. Venn diagram and drug-targets-disease network of Rg1 targets and PD-related genes. There were 100 Rg1 targets and 9,366 PD-related genes, 72 of which were common targets. PPI network and molecular docking of HSP900AA1 and MMP9. The PPI network contained 67 key targets. The binding energy of HSP900AA1 and MMP9 were −8.6 kcal/mol and −7.1 kcal/mol, respectively, indicating a good docking effect.

There exists a substantial obstacle in bringing Rg1 to clinical practice due to the requirement to demonstrate consistent research results that apply to the broad patient population. Animal trials and initial human clinical data form the basis for current research, generating doubts about their relevance when applied to large human clinical trials. The intricate study on the mechanism of action of Rg1 reveals valuable therapeutic possibilities, yet constituting obstacles for researchers trying to design success studies. Ironically, there is still an absence of complete assessments around potential unintended side effects related to the extensive target diversity of the action. Upcoming studies on well-controlled randomized trials are needed to profile the effectiveness of Rg1 among different populations, and develop dose and treatment guidelines. Network pharmacology can be employed to identify related pathways explaining the operational mechanism of Rg1, functioning as an effective tool. Through this approach, potential molecular targets can be identified systematically, which grants researchers better insight into therapeutic opportunities of Rg1. We will continue to elaborate network pharmacology outcomes, aiming at creating stronger scientific support for employing Rg1 in clinical practice. Network pharmacology techniques enable the location of possible targets of Rg1 within PD (PD). First, each possible target for Rg1 could be determined by using the SwissTargetPrediction tool (Our study used SwissTargetPrediction (http://www.swisstargetprediction.ch/). Next, genes related to PD could be filtered using the GeneCards database (https://www.genecards.org/). These targets were then imported into Cytoscape 3.10 to establish a drug-target-disease network, followed by the construction of a protein-protein interaction (PPI) network using String database (https://cn.string-db.org/). The PPI network was useful for the selection of targets for further analysis, representing areas with established experimental support. Furthermore, to verify the reliability of these targets, molecular docking was performed using AutoDockTools 1.5 and PyMOL, in combination with the PDB database (https://www.rcsb.org/). Finally, Rg1 presented 100 potential targets, which matched with a set of 9,366 PD-related targets to produce 72 shared targets for examination. Among these, HSP90AA1 and MMP9 were enrolled for thorough molecular docking analysis. AutoDockTools determined the binding energies to be −8.6 kcal/mol for HSP90AA1 and −7.1 kcal/mol for MMP9, as depicted in Figure 2. Our study demonstrated definitive binding relationships between targets, validating both their trustworthiness and potential use in therapy. We deployed network pharmacology to systematically map potential targets and biological pathways of Rg1, while developing interconnected drug-target-disease networks, for a purpose of better understanding molecular multi-target actions. Significantly, merging bioinformatics data with this technique can speed up target validation, resulting in more efficient research progress. Molecular docking analysis validates the identified targets, delivering strong scientific proof of the neuroprotective abilities of Rg1.

## Conclusion

Rg1 has shown significant promise for the treatment of PD in both foundational and clinical research, primarily attributed to its robust neuroprotective effects, such as its anti-inflammatory and antioxidant properties. Rg1 can effectively alleviate PD-associated motor and cognitive dysfunctions through various mechanisms, including the inhibition of neuroinflammation, reduction of oxidative stress, promotion of neuronal survival, and enhancement of synaptic plasticity. At this stage, the therapeutic efficacy of Rg1 remains to be established by further clinical validation, although the initial findings are encouraging. In terms of future direction of investigation, we can continue to comprehensively evaluate the long-term safety and effectiveness of Rg1 through large-scale, multi-center clinical trials; improve the bioavailability and targeting accuracy of Rg1 using advanced technologies like nanotechnology; explore potential synergies with current treatments to optimize therapeutic outcomes and minimize adverse effects; and conduct a deeper analysis of the molecular pathways that mediate the effects of Rg1 to fully understand its role in neurodegenerative disease treatment. Continued research can position Rg1 as a valuable therapeutic option for PD, offering improved therapeutic solutions for patients.
